# The Role of Autoantibodies in Arrhythmogenesis

**DOI:** 10.1007/s11886-020-01430-x

**Published:** 2020-11-25

**Authors:** Jin Li

**Affiliations:** 1 Institute of Biochemistry and Molecular Medicine, University of Bern, Bühlstrasse 28, 3012 Bern, Switzerland; 2 Department of Cardiology, Lausanne University Hospital, rue du Bugnon 46, 1011 Lausanne, Switzerland

**Keywords:** Atrial fibrillation, Autoantibodies, Autoimmunity, Cardiac arrhythmias, Cardiac conduction disease, Ventricular arrhythmia

## Abstract

**Purpose of Review:**

The role of autoantibodies in arrhythmogenesis has been the subject of research in recent times. This review focuses on the rapidly expanding field of autoantibody-mediated cardiac arrhythmias.

**Recent Findings:**

Since the discovery of cardiac autoantibodies more than three decades ago, a great deal of effort has been devoted to understanding their contribution to arrhythmias. Different cardiac receptors and ion channels were identified as targets for autoantibodies, the binding of which either initiates a signaling cascade or serves as a biomarker of underlying remodeling process. Consequently, the wide spectrum of heart rhythm disturbances may emerge, ranging from atrial to ventricular arrhythmias as well as conduction diseases, irrespective of concomitant structural heart disease or manifest autoimmune disorder.

**Summary:**

The time has come to acknowledge autoimmune cardiac arrhythmias as a distinct disease entity. Establishing the autoantibody profile of patients will help to develop novel treatment approaches for patients.

## Introduction

**Fig. 1:**
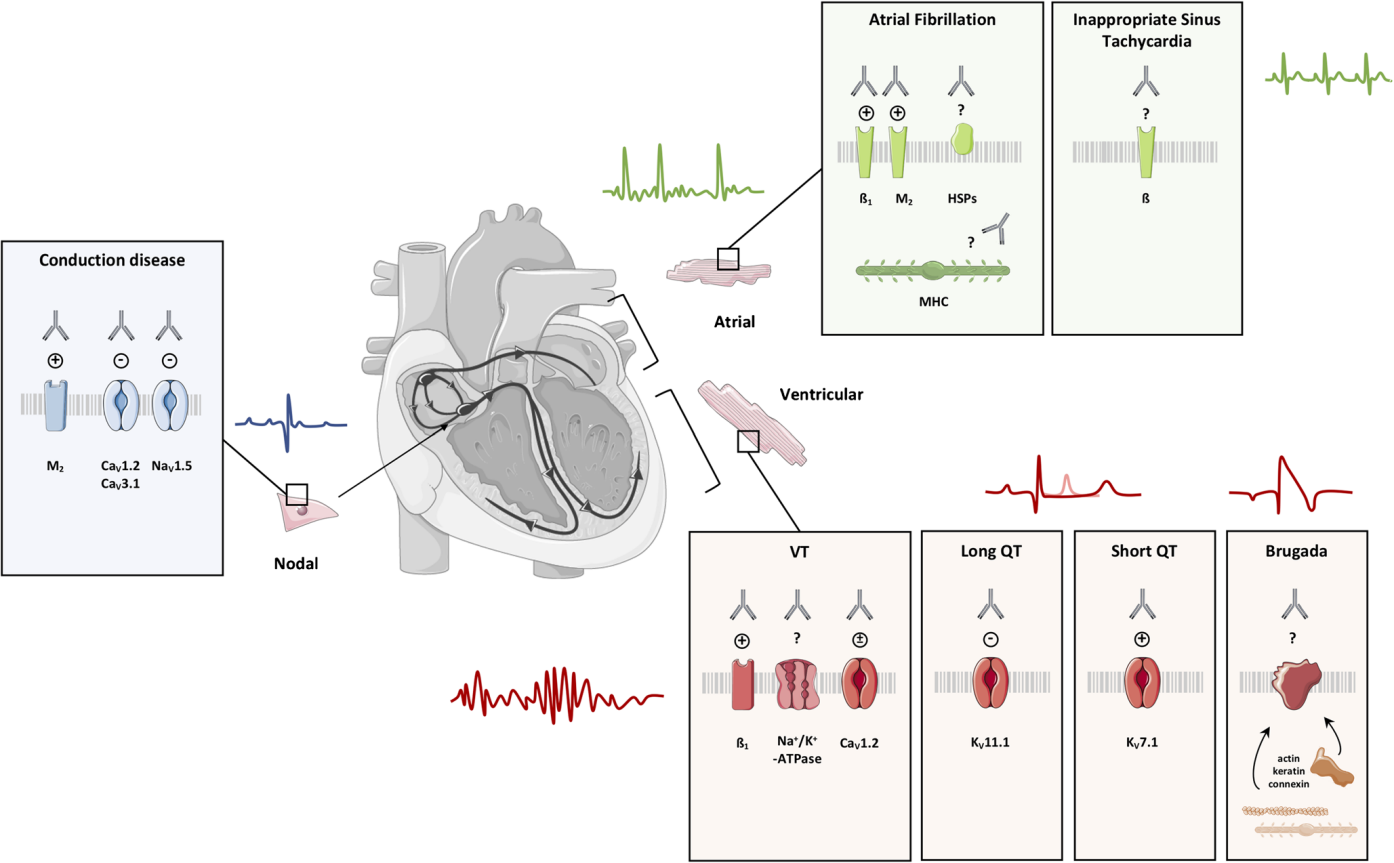
Summary of autoantibodies related to atrial fibrillation, inappropriate sinus tachycardia, conduction diseases and ventricular arrhythmias, identified so far. ß ß-adrenergic receptor, Ca_v_1.2 L-type voltage-gated Ca^2+^ channel, Ca_v_3.1 T-type voltage-gated Ca^2+^ channel, HSP heat shock protein, K_v_7.1 voltage-gated KCNQ1 K^+^ channel, K_v_11.1 voltage-gated KCNH2 K^+^ channel, M_2_ M_2_-muscarinic acetylcholine receptor, Na_v_1.5 voltage-gated Na^+^ channel, VT ventricular tachyarrhythmia, ± stimulation/inhibition. This image was produced using images modified from Servier Medical Art

With the conceptualization of the contradictory capacity of the immune system to self-defend and self-destruct at the same time, the term autoimmunity was first coined over a century ago [1•, 2]. Beyond the philosophical implication, this vital paradox shaped our present-day understanding of disease development when self-tolerance is lost and autoantibodies considered a sine qua non of the condition [1•, 3]. The contribution of autoimmunity in cardiovascular diseases in general is largely under-recognized, even more in the context of heart rhythm disturbances. While anatomical features and genetic background were classically seen as the substrates for cardiac arrhythmias, in recent years, it has become increasingly clear that functional autoantibodies can induce arrhythmias by interfering with ion channels and receptors, the key determinants of cardiac electrophysiology. This review focuses on autoantibody-mediated cardiac arrhythmias, classifying them in three sections, according to their origin: atrial, nodal, and ventricular (Fig. 1). Table 1 summarizes the current literature on autoantibody-induced ECG abnormalities, the prevalence, and associated clinical features.

**Table 1:** Autoantibodies and cardiac arrhythmias

ECG abnormalities	Clinical features	Prevalence	Target antigen	EP mechanism
Atrial arrhythmias				
AF	Adults, no structural heart disease	60% [4]	Myosin heavy chain	n/a
AF	Adults, no structural heart disease	23% [5]	M_2_-muscarinic acetylcholine receptor	↑ *I*_K,Ach_
AF	Adults, no structural heart disease	n/a [6]	ß_1_-adrenergic receptor	n/a
AF	Adults, coronary artery disease	n/a [7]	Heat shock protein 65	n/a
AF	Adults, coronary artery disease	n/a [8]	Heat shock protein 60	n/a
AF	Adults, no structural heart disease	21% [9]	Heat shock protein 70	n/a
Inappropriate sinus tachycardia	Adults, no structural heart disease	52% [10]	ß-adrenergic receptor	n/a
Nodal arrhythmias				
SA + AV block	In utero until age 27 days	2–5% [11••, 12, 13]	Ro/SSA, La/SSB, Ca_v_1.2 (CACNA1c), Ca_v_3.1 (CACNA1g)	↓ *I*_Ca,L_ and *I*_*Ca,T*_
AV block III°	Adults, no structural heart disease	10% [14]	Ro/SSA, Ca_v_1.2 (CACNA1c), Ca_v_3.1 (CACNA1g)	↓ *I*_Ca,L_ and *I*_*Ca,T*_
SND	Adults, no structural heart disease	75% [15]	M_2_-muscarinic acetylcholine receptor	↑ *I*_K,Ach_
SND	Adults, dilated cardiomyopathy	18–51% [16]	M_2_-muscarinic acetylcholine receptor	↑ *I*_K,Ach_
SND	Adults, Chagas’ disease	40–77% [16, 17]	M_2_-muscarinic acetylcholine receptor	↑ *I*_K,Ach_
AV block	Adults, no structural heart disease	n/a [18]	Na_v_1.5 (SCN5A)	↓ *I*_Na_
Ventricular arrhythmias				
PVC + VT	Adults, no structural heart disease	48–72% [15, 19, 20]	ß_1_-adrenergic receptor	↑ *I*_Ca,L_,↑ *I*_Kr_, ↓*I*_Ks_
PVC + VT	Adults, dilated cardiomyopathy	26% [21]	Na^+^/K^+^-ATPase	n/a
VT	Adults, dilated cardiomyopathy	49% [22, 23]	Ca_v_1.2 (CACNA1c), N-terminus	↑ *I*_Ca,L_
VT	Adults, no structural heart disease	71% [24••]	Ca_v_1.2 (CACNA1c), pore domain	↓ *I*_Ca,L_
Long QT	Adults, no structural heart disease	10–60% [11••, 25–32]	Ro/SSA, K_v_11.1 (KCNH2, *h*ERG)	↓ *I*_Kr_
Short QT	Adults, dilated cardiomyopathy	6% [33]	K_v_7.1 (KCNQ1, KvLQT1)	↑ *I*_Ks_
Brugada pattern	Adults, Brugada syndrome	100% [34]	α-cardiac actin, α-skeletal muscle actin, keratin-24, connexin-43	n/a

*AF* atrial fibrillation, *AV* atrioventricular, *Ca*_*v*_*1.2* L-type voltage-gated Ca^2+^ channel, *Ca*_*v*_*3.1* T-type voltage-gated Ca^2+^ channel, *EP* electrophysiological, *hERG* human ether-à-go-go-related gene, *I*_*Ca,L*_ L-type voltage-gated Ca^2+^ current, *I*_*Ca,T*_ T-type voltage-gated Ca^2+^ current, *I*_*K,Ach*_ acetylcholine-regulated K^+^ current, *I*_*Kr*_ rapidly activating delayed rectifier K^+^ current, *I*_*Ks*_ slowly activating delayed rectifier K^+^ current, *I*_*Na*_ voltage-gated Na^+^ current, *K*_*v*_*7.1 KvLQT1* voltage-gated KCNQ1 K^+^ channel, *K*_*v*_*11.1* voltage-gated KCNH2 K^+^ channel, *Na*_*v*_*1.5* voltage-gated Na^+^ channel, *n/a* not available, *PVC* premature ventricular complex, *SA* sinoatrial, *SND* sinus node dysfunction, *VT* ventricular tachyarrhythmia

## Autoantibodies and Atrial Arrhythmias

The first evidence supporting a possible role of autoantibodies in the development of atrial arrhythmias derived from a small-scale study describing anti-myosin heavy chain autoantibodies in 60% of patients with atrial fibrillation (AF) [3, 4]. Because growing evidence supports the significance of the cardiac autonomic nervous system in AF development, conceptually, it makes sense that binding of functional autoantibodies to G protein-coupled receptors be associated with AF [35]. As parasympathomimetic and sympathomimetic agonists, respectively, anti-M_2_-muscarinic acetylcholine and anti-ß_1_-adrenergic receptor autoantibodies, were naturally presumed to contribute to AF pathogenesis. In fact, several studies demonstrated that anti-M_2_-muscarinic acetylcholine and anti-ß_1_-adrenergic receptor autoantibodies are independent predictors of AF in patients with no underlying structural heart disease [5, 6, 36]. Moreover, studies have shown that anti-M_2_-muscarinic acetylcholine and anti-ß_1_-adrenergic receptor autoantibodies can be used as predictive markers of AF recurrence 1 year after ablation therapy [37, 38]. Animal studies addressing the pathophysiological mechanism have shown the potential of anti-M_2_-muscarinic acetylcholine and anti-ß_1_-adrenergic receptor autoantibodies to induce atrial structural (fibrosis) and electrophysiological remodeling (increased anti-M_2_-muscarinic acetylcholine receptor-*I*_K,Ach_ pathway, atrial effective refractory period shortening), thus forming the underlying substrate for AF (increased atrial arrhythmogenicity) [5, 39–42]. Nevertheless, the role of anti-M_2_-muscarinic acetylcholine and anti-ß_1_-adrenergic receptor autoantibodies as mere bystander, biomarker, or pathogen in AF is being scrutinized, and the causal relationship remains a matter of ongoing debate [3, 43]. Heat shock proteins (HSPs) are intracellular chaperones that help to preserve cellular integrity through proper protein folding and conformation [43]. In response to stress, HSPs are translocated to the plasma membrane and thus present potential targets for circulating autoantibodies [43]. Accordingly, invasive procedures such as cardiac surgery expose cardiomyocytes to stressful stimuli and presumably induce the production of anti-HSP autoantibodies [7, 8]. Anti-HSP65 and anti-HSP60 autoantibodies have been reported in post-operative AF, while anti-HSP70 autoantibodies are associated with AF recurrence post-ablation therapy [7–9]. At present, the role of anti-HSP autoantibodies in the pathogenesis of AF remains unclear.

Inappropriate sinus tachycardia is a diagnosis of exclusion and manifests as unexpectedly elevated resting heart rate and/or disproportionate heart rate response to physical activities, in a structurally normal heart [3, 44]. The etiology has yet to be elucidated, but one study explored the link between inappropriate sinus tachycardia and anti-ß-adrenergic receptor autoantibodies [10]. Interestingly, anti-ß-adrenergic receptor autoantibodies were identified in half of the patients and accounted for the positive chronotropic effect on rat cardiomyocytes through stimulation of the ß-adrenergic receptor [10].

## Autoantibodies and Nodal Arrhythmias

It was in 1976 when Fairfax and Doniach first described the existence of autoantibodies targeting the cardiac conduction tissue in patients with left bundle branch block [45]. Barely a decade later, the involvement of autoantibodies in cardiac conduction disturbances has evolved substantially [11••, 46]. Anti-Ro/SSA and anti-La/SSB immunoglobulins (named after the patients’ name they were extracted from/Sjögren’s Syndrome autoantigen A and B, respectively) are the archetypal autoantibodies in arrhythmogenesis. Best known in the context of autoimmune connective tissue disorders, the Ro/SSA and La/SSB antigens are intracellular ribonucleoproteins to which autoantibodies are found in correlation with perinatal cardiac conduction disturbances [11••]. Anti-Ro/SSA and anti-La/SSB positive mothers have a 2–5% risk of delivering an infant with congenital heart block (CHB), a disease spectrum encompassing conduction abnormalities affecting the sinoatrial (SA) and atrioventricular (AV) nodes of fetuses and neonates [11••, 12, 13]. While resolution of sinus bradycardia and lower-degree AV block is generally observed either spontaneously or after maternal immunosuppressive therapy (steroids, plasmapheresis and/or intravenous immunoglobulin), third-degree AV block is irreversible [11••, 47–50]. Anti-Ro/SSA may react with two different subtypes of the Ro antigen, referred to as anti-Ro/SSA-52kD and anti-Ro/SSA-60kD according to the different molecular weights. Anti-Ro/SSA-52kD has become the main focus of interest with its predominant role in CHB. Numerous studies on animals (ranging from murine to rat, guinea pig, and rabbit models) as well as fetal human hearts have provided evidence that anti-Ro/SSA antibodies from sera of mothers lead to CHB through transplacental passage as early as 11 weeks of gestational age and cross-reaction with the Ca^2+^ channels (L- and T-type) of the fetal cardiac conduction system [11••]. The subsequent inhibition of Ca^2+^ currents (*I*_Ca,L_ and *I*_Ca,T_) disrupts the pulse generation and propagation in SA and AV nodes [11••]. Furthermore, the chronic exposure to anti-Ro/SSA downregulates the surface expression of L-type Ca^2+^ channels resulting in apoptotic cell death and triggering inflammation [11••]. This process ultimately leads to fibrosis and calcification of the conduction system, characteristic of CHB [11••, 51]. Because CHB does not develop systematically in all anti-Ro/SSA-positive pregnancies, the involvement of other autoantibodies has been suggested including autoantibodies targeting calreticulin, the M_1_-muscarinic acetylcholine receptor, α-fodrin, α-enolase, serotoninergic 5-hydroxytryptophane (5-HT_4_) receptor, and endogenous retrovirus-3 placental protein [13, 46, 52–58]. However, investigations on these autoantibody candidates are limited to case studies. Their underlying mechanism and clinical relevance remain uncertain [46].

In adults, the association between anti-Ro/SSA autoantibodies and cardiac conduction disturbances is less evident [11••, 59]. Unlike the fetal heart, the expression of L- and T-type Ca^2+^ channels is higher in adults, rendering them more resistant to anti-Ro/SSA-induced Ca^2+^ current inhibition [11••, 59]. Nevertheless, based on a retrospective study, it is estimated that about 10% of adults with isolated third-degree AV block is related to anti-Ro/SSA autoantibodies [14]. Another autoantibody associated with cardiac conduction disease targets the M_2_-muscarinic acetylcholine receptor. It has been detected in 75% of patients with primary sinus node dysfunction and was more commonly reported in dilated cardiomyopathy and Chagas’ disease with sinus node dysfunction [3, 15–17]. Anti-M_2_-AChR autoantibodies presumably act as agonists on the M_2_-muscarinic acetylcholine receptor, which subsequently inhibits the Ca^2+^ current (*I*_Ca,L_) and activates the acetylcholine-regulated K^+^ current (*I*_K,ACh_) of SA nodes. Finally, one study detected for the first time, autoantibodies targeting the cardiac voltage-gated Na^+^ channel (Na_V_1.5, SCN5A) in patients with idiopathic high-degree AV block (second-degree AV block Mobitz type II and third-degree AV block) [18]. Because the autoantibody screening was performed in the pooled serum of 10 patients, the prevalence of anti-Na_v_1.5 autoantibody-mediated AV block cannot be determined. The serum of these patients with conduction disease reduced the Na^+^ current (*I*_Na_) density in rat cardiomyocytes compared to the serum of healthy controls [18]. Moreover, rats with anti-Na_v_1.5 autoantibodies consistently developed intermittent third-degree AV block and SA block [18]. The proposed mechanism is a downregulation of Na_v_1.5 channel expression leading to *I*_Na_ reduction, an impairment that the AV nodal region is particularly vulnerable to, owing to the localized lower Na^+^ channel density [18].

## Autoantibodies and Ventricular Arrhythmias

Disruption of cardiac repolarization appears to be the common electrophysiological pathway of autoantibodies leading to ventricular arrhythmias.

Autoantibodies targeting ß_1_-adrenergic receptors were described in a variety of cardiac diseases including dilated cardiomyopathy (26–95%), ischemic cardiomyopathy (10–55%), and Chagas’ disease (30–98%) [60, 61••]. Soon after discovering their sympathomimetic effect, it became clear that their contribution to arrhythmogenesis was not limited to structural remodeling process (e.g., myocardial hypertrophy, ventricular dilatation, and dysfunction). Anti-ß_1_-adrenergic receptor autoantibodies were reported in 48–72% of patients with clinical signs of ventricular electrical instabilities, ranging from frequent premature ventricular complexes (PVCs) to sustained ventricular tachyarrhythmias (VTs), despite a structurally normal heart [15, 19, 20]. The suggested mechanism of anti-ß_1_-adrenergic receptor autoantibody-mediated ventricular arrhythmias is ß-adrenergic stimulation leading to ion channel remodeling including adrenergically enhanced inward *I*_Ca,L_ and increased rapidly activating delayed rectifier K^+^ current (*I*_Kr_) along with reduced slowly activating delayed K^+^ current (*I*_Ks_) [62, 63]. The net effect is an abnormally prolonged cardiac repolarization, the substrate for lethal arrhythmias.

During activation of the sympathetic nervous system, Na^+^/K^+^-ATPase is an integral player of the adrenergic response [64]. In this context, one study detected autoantibodies directed against Na^+^/K^+^-ATPase in 26% of patients with dilated cardiomyopathy [21]. Anti-Na^+^/K^+^-ATPase autoantibody-positive patients presented more frequently PVCs and non-sustained VTs [21]. After 31 months of follow-up, the presence of anti-Na^+^/K^+^-ATPase autoantibodies was an independent predictor of sudden cardiac death [21]. The exact pathomechanism is not known, but an impaired Ca^2+^ signaling cascade is suspected [21].

Pertaining to Ca^2+^ handling abnormalities, autoantibodies targeting the N-terminus of the L-type voltage-gated Ca^2+^ channel (Ca_v_1.2, α1c pore-forming subunit or CACNA1c) are present in 49% of patients with dilated cardiomyopathy [22]. The autoantibody was an independent predictor of VT and sudden cardiac death after a mean follow-up period of 32 months [22]. Experimental studies demonstrated an autoantibody-mediated prolongation of action potential duration (APD) and induction of early afterdepolarizations (EADs) through enhancement of *I*_Ca,L_ [22, 23]. In addition, anti-Ca_v_1.2 autoantibodies induced VT in rat hearts [22]. And yet, how the autoantibody interacts with an intracellular epitope of the Ca^2+^ channel remains unknown [22, 23]. Only recently, a novel autoantibody targeting an extracellular domain of the cardiac Ca^2+^ channel (α1c, Ca_v_1.2) was discovered and related to VT underlying sudden cardiac arrest in patients without any structural heart disease [24••]. Functional studies demonstrated the proarrhythmogenic effect of anti-Ca_v_1.2 autoantibodies in human-induced pluripotent stem cell-derived cardiomyocytes through inhibition of Ca_v_1.2 channels and subsequent APD shortening [24••]. Given the close proximity of the target site to the pore of the channel, it is speculated that alteration of the ion selectivity and permeability filter predisposes to ventricular arrhythmias [24••].

Until recently, it was believed that the effect of anti-Ro/SSA autoantibodies was confined to cardiac Ca^2+^ channels as described in the previous section. However, since at least the emblematic case report of a seemingly healthy woman presenting with Torsades-de-pointes (TdP) ventricular arrhythmias, we know that anti-Ro/SSA autoantibodies can cause a substantial delay in cardiac repolarization [65]. Anti-Ro/SSA autoantibodies reduce the repolarizing *I*_Kr_ current through direct inhibition of the *h*ERG channel (human ether-à-go-go-related gene, K_v_11.1 or KCNH2), which results in APD lengthening and QT_c_ prolongation [11••, 65]. In fact, the targeted epitope of the *h*ERG channel shares 44% sequence homology with the Ro/SSA-52kD antigen, thus advocating the binding of anti-Ro/SSA autoantibodies with the channel [11••]. Retrospectively, several studies have previously suggested a link between anti-Ro/SSA autoantibodies and QT_c_ prolongation in patients with autoimmune connective tissue diseases, while others found no correlation [11••, 25–32, 66–68]. Differences in autoantibody titer and subtype specificity may account for the variability in the prevalence of prolonged QT_c_, ranging from 10 to 60% [11••, 25–32]. Notably the anti-Ro/SSA-52kD subtype is held responsible for the observed proarrhythmogenic effects on the ventricles, as evidenced by Lazzerini et al. [32]. In support of the notion of autoimmune-associated long QT syndrome (LQTS), anti-Ro/SSA autoantibodies are found in 60% of patients with TdP, mostly in the absence of any history of autoimmune disease [32].

The functional counterpart of LQTS is short QT syndrome (SQTS), characterized by an abnormally shortened ventricular repolarization secondary to a cardiac ion channel dysfunction. Traditionally, a genetic mutation is the underlying cause. In light of the emerging field of autoimmune-mediated cardiac arrhythmias, the first form of autoantibody-induced SQTS was recently identified [33]. Autoantibodies targeting the voltage-gated KCNQ1 K^+^ channel (K_v_7.1 or K_v_LQT1) forming *I*_Ks_ was detected in 6% of patients with dilated cardiomyopathy and associated with a significantly shorter QT_c_ interval [33]. The findings were reproducible in an experimental animal model immunizing rabbits with the target KCNQ1 peptide sequence [69]. In agreement with the clinical data, rabbits with KCNQ1 autoantibodies had a shorter QT_c_ interval on ECG, shortened ventricular effective refractory period, and increased susceptibility to VT upon programmed ventricular stimulation [69]. Mechanistically, anti-KCNQ1 autoantibodies increase the open time and open probability of KCNQ1 channels [70••]. The resulting enhanced *I*_Ks_ current shortens the ventricular repolarization phase [70••].

Brugada syndrome constitutes a further primary arrhythmia syndrome next to LQTS and SQTS. It is widely accepted that a genetic mutation in a cardiac ion channel is the main cause of the condition, although structural changes and inflammatory processes have been attributed a pathogenic role [34]. Autoantibodies targeting α-cardiac actin, α-skeletal muscle actin, keratin-24, and connexin-43 have now been revealed as biomarkers of Brugada syndrome [34]. The functional role of these autoantibodies is not clear, and further studies will be needed to clarify the pathomechanisms underlying the autoimmune response [34].

## Conclusions

The role of autoantibodies in arrhythmogenesis has long been overlooked but autoantibody-mediated arrhythmias are now beginning to be widely recognized as a distinct disease entity. As part of this paradigm shift, cardiac arrhythmias once classified as “idiopathic” need to be reappraised. The recognition of the autoimmune etiology is not simply a matter of terminology; it is of major importance for the management of patients. Immunosuppressive measures including immunomodulatory drugs and plasmapheresis could successfully treat patients with anti-Ro/SSA-mediated AV block [71–73]. Immunoadsorption for autoantibody removal has been effectively employed for the treatment of ß_1_-adrenergic receptor autoantibody-positive patients with dilated cardiomyopathy [1••]. Furthermore, peptides have been designed to neutralize the binding of pathogenic autoantibodies. Preliminary in vitro data are encouraging, but clinical studies will be needed to confirm the therapeutic potential [1••, 11••, 22, 24••]. Finally, the discovery of an autoantibody, pathogenic for one, may actually shape up as promising new treatment approach for another. Immunotherapy for LQTS type 2 is the prototypic example [69, 70••]. Through *I*_Ks_ upregulation, anti-KCNQ1 antibodies have the potential to compensate for the loss of *I*_Kr_ as in LQTS type 2 [70••]. Accordingly, anti-KCNQ1 antibodies from active immunization (KCNQ1 peptide vaccination) have been shown effective for the treatment of acquired LQTS type 2 in rabbits, while passive immunization (KCNQ1 antibody therapy) was found therapeutic in a cellular model of congenital LQTS type 2 [69, 70••]. Apart from being either a biomarker or a pathogen, natural autoantibodies exist in the healthy and are considered essential for the physiological tissue homeostasis [70••]. A recent comprehensive screening for autoantibodies targeting cardiac ion channels best illustrates the complexity of autoantibodies in healthy individuals [70••]. Their abundance and diversity imply that they may harbor some important function yet to be unveiled [70••]. Just as any puzzle solvers know, finding the corner pieces is a first step to the complete picture. While autoantibodies constitute only one such corner; inflammatory cytokines and immune cells are further pieces of the autoimmune arrhythmia puzzle [74•, 75•]. This exciting area of research in cardioimmunology is rapidly expanding, and putting together the puzzle will turn our understanding of the pathogenesis into a means for developing novel treatments of cardiac arrhythmias, becoming ever more efficient and personalized.
